# Improving signal strength in serial crystallography with *DIALS* geometry refinement

**DOI:** 10.1107/S2059798318009191

**Published:** 2018-09-03

**Authors:** Aaron S. Brewster, David G. Waterman, James M. Parkhurst, Richard J. Gildea, Iris D. Young, Lee J. O’Riordan, Junko Yano, Graeme Winter, Gwyndaf Evans, Nicholas K. Sauter

**Affiliations:** a Lawrence Berkeley National Laboratory, Berkeley, CA 94720, USA; b STFC Rutherford Appleton Laboratory, Didcot OX11 0QX, England; c CCP4, Research Complex at Harwell, Rutherford Appleton Laboratory, Didcot OX11 0FA, England; d Diamond Light Source Ltd, Harwell Science and Innovation Campus, Didcot OX11 0DE, England; e MRC Laboratory of Molecular Biology, Francis Crick Avenue, Cambridge CB2 0QH, England

**Keywords:** XFEL, metrology, *DIALS*, refinement, sparse algebra

## Abstract

The *DIALS* diffraction-modeling software package has been applied to serial crystallography data. Diffraction modeling is an exercise in determining the experimental parameters, such as incident beam wavelength, crystal unit cell and orientation, and detector geometry, that are most consistent with the observed positions of Bragg spots. These parameters can be refined by nonlinear least-squares fitting. In previous work, it has been challenging to refine both the positions of the sensors (metrology) on multipanel imaging detectors such as the CSPAD and the orientations of all of the crystals studied. Since the optimal models for metrology and crystal orientation are interdependent, alternate cycles of panel refinement and crystal refinement have been required. To simplify the process, a sparse linear algebra technique for solving the normal equations was implemented, allowing the detector panels to be refined simultaneously against the diffraction from thousands of crystals with excellent computational performance. Separately, it is shown how to refine the metrology of a second CSPAD detector, positioned at a distance of 2.5 m from the crystal, used for recording low-angle reflections. With the ability to jointly refine the detector position against the ensemble of all crystals used for structure determination, it is shown that ensemble refinement greatly reduces the apparent nonisomorphism that is often observed in the unit-cell distributions from still-shot serial crystallography. In addition, it is shown that batching the images by timestamp and re-refining the detector position can realistically model small, time-dependent variations in detector position relative to the sample, and thereby improve the integrated structure-factor intensity signal and heavy-atom anomalous peak heights.

## Abstract 2

For XFEL data, simultaneous refinement of multi-panel detector geometry with thousands of crystal models in the program *DIALS* improves the integrated signal quality and helps to reduce non-isomorphism

## 1. Introduction   

Serial crystallographic methods are widening the scope of structural biology, allowing the examination of macromolecular structure with short radiation pulses that generate diffraction from samples nearly free of radiation damage. Room-temperature experiments preserve the physiologically relevant dynamic motion of proteins that cryopreservation quenches and can span multiple time points along enzymatic pathways. Present-day developments began with the introduction of X-ray free-electron laser (XFEL) sources (Bergmann *et al.*, 2017 ▸); however, the latest generation of synchrotron-radiation sources have introduced pulse durations and focus sizes that confer some of the same benefits.

While groundbreaking in its promise, serial crystallography presents numerous technical challenges, including those involving data analysis. With short X-ray pulses, ranging from microseconds at synchrotrons to femtoseconds at XFELs, crystalline specimens are essentially still for the duration of a single shot, after which the crystal is replaced. This contrasts with conventional methods, in which a single crystal is continually rotated on a goniometer. The data-processing workflows are broadly similar for both methods, consisting of the location of Bragg spots by a spot-finding algorithm, the determination of the crystal lattice by an indexing procedure, the integration of diffraction intensities at predicted Bragg spot positions, and finally the scaling and merging of repeated Bragg measurements. Also, in both cases the approach involves inverse modeling, in which a computer representation of the experiment is used to predict properties of the diffraction image, including the Bragg spot positions, after which the parameters of the model are iteratively adjusted to best match the observed images.

However, while the treatment of rotation shots has been well established for several decades, experimental innovations in serial crystallography have required new models. XFEL facilities, in particular, introduced pixel-array detectors that are uniquely designed to integrate X-ray signals over periods of femtoseconds. These carry the performance tradeoff of being constructed as multipanel units, with the added issue that the geometrical relationship between the individual panels (the ‘metrology’) must be included in the computational model. Our group (Hattne *et al.*, 2014 ▸) and others (Yefanov *et al.*, 2015 ▸; Ginn & Stuart, 2017 ▸) have shown that the positions and orientations of individual detector panels, which are poorly determined initially, can be determined to subpixel accuracy by iterative nonlinear least-squares refinement, which minimizes the residual difference between observed and predicted Bragg spot positions.

**Figure 1:**
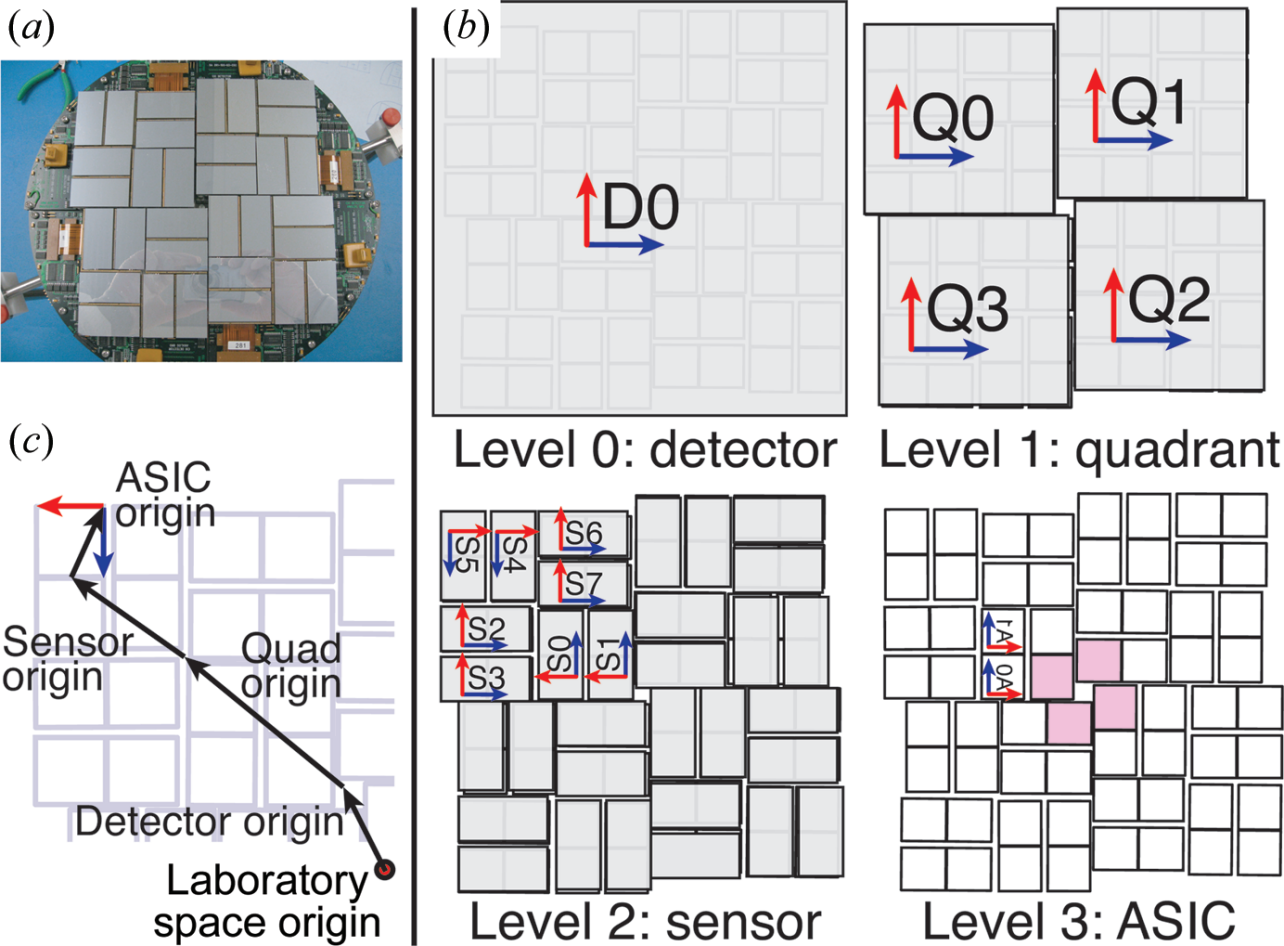
Overview of the CSPAD (Cornell–SLAC Pixel Array Detector). (*a*) Photograph of the instrument (credit: Philip Hart). (*b*) Hierarchical organization. Each level groups objects that can be refined together. The pink ASICs in level 3 are used with powder patterns for quadrant alignment (§[Sec sec3.2]3.2). The blue and red vectors are the **d**_*x*_ and **d**_*y*_ directions used to orient the group. The **d**_*n*_ vector completing the coordinate system is orthogonal to both, pointing out of the page for levels 0–2. At level 3, a *y*-axis sign flip is used to align the fast (blue) and slow (red) directions used to read out the pixels from the raw data, which also flips **d**_*n*_ to point into the page (note the inverted ‘A0’ and ‘A1’ labels). (*c*) Origin vectors **d**_*n*_ for each level of the CSPAD hierarchy. Starting at the origin of laboratory space, the detector is shifted by the detector origin vector. The deeper hierarchy levels point from the parent object origin to the child object origins, or in the case of the ASICs to the position of the (0, 0) pixel. Note that it might be expected that quadrants 1–3 would be rotated 90, 180 and 270° clockwise, respectively, and that S6 and S7 would be rotated 180°, all to maintain the fourfold symmetry of the detector. However, how the metrology is converted from optical measurements to vectors is arbitrary and varies each time the CSPAD is reassembled, sometimes producing a quadrant pattern without fourfold symmetry. Such is the case for the L785 experiment illustrated here. *DIALS* handles arbitrary configurations, so this is not an issue.

Despite the success of the Ha14 (Hattne *et al.*, 2014 ▸) metrology code included with our program *cctbx.xfel*, it has been understood that the implementation would eventually need to be redesigned for several reasons. Ha14 embeds the image data from all detector panels into a single square-shaped data array that represents the whole detector, with panels in their approximate geometric positions, against a backdrop of pixels that are set to a special value to signify the inactive areas between panels. Superficially, this is open to the criticism that the inactive areas waste memory and disk space. However, the disadvantage has proven to be more severe owing to the necessity of encoding the number of panels and their dimensions, as well as the need for special code to ignore the inactive pixels for image processing. As a result, while the code supports the 64-panel CSPAD (Fig. 1 ▸; Hart *et al.*, 2012 ▸) installed at the Linac Coherent Light Source (LCLS) at the time of the Ha14 publication, it cannot be easily adapted for use with subsequent detector models, including the eight-panel MPCCD detector at SACLA (Kameshima *et al.*, 2014 ▸) and the 128-panel AGIPD detector at the European XFEL (Henrich *et al.*, 2011 ▸). Even the temporary loss of a single sensor on the CSPAD detector requires the reduced sensor complement to be hard-coded.

Several additional considerations led us to abandon the single-array approach to data representation. Firstly, the Ha14 design unnecessarily conflates the concepts of measurement and model. For example, if we determine after data collection that our model should move one of the sensors two pixels to the right, a new copy of the data array has to be created to reflect the updated sensor position. Furthermore, the single-array approach does not allow the possibility that the distances between sensors can assume fractional pixel values or that the sensors might be slightly rotated with respect to each other. Thus, the Ha14 code is forced to maintain a separate data structure that encodes corrections to the unit-pixel metrology. A better software design, adopted here, is to maintain two data structures, one that simply contains the original detector-panel measurements in their unaltered forms (as a list of rectangular sensor arrays of pixels) and another that represents the complete vector description of each panel, including the origin vector **d**_0_ that locates the panel in relation to the crystal and two vectors **d**_*x*_ and **d**_*y*_ that define the fast and slow readout directions (Parkhurst *et al.*, 2014 ▸). This approach also removes the undesirable requirements in Ha14 that all detector panels are coplanar and that the plane of the detector is normal to the beam.

There is also good reason to reorganize our geometric description of a multipanel detector (referred to below as simply the ‘detector model’) with a hierarchical design that mirrors the physical construction of the device (Brewster *et al.*, 2014 ▸). For the CSPAD detector in particular (Fig. 1 ▸), we assign four levels of organization, with the overall detector composed of four separately constructed quadrants, each of which in turn is composed of eight silicon-wafer sensors. The silicon sensors are bump-bonded to two 194 × 185 pixel ASIC (application-specific integrated circuit) arrays (Hart *et al.*, 2012 ▸). Model elements at each level contain **d**_0_ vectors expressed relative to the next highest level (Fig. 1 ▸). The LCLS facility has the capability of determining the sensor positions within each quadrant at the time of assembly to pixel-level accuracy, using optical microscopy methods. Expressing our detector model as a hierarchy allows us to insert the LCLS quadrant-level calibrations at the appropriate level, to be used as starting values for the least-squares refinement, with the main uncertainties therefore being the interrelationship of the four quadrants and the position of the detector as a whole. Additionally for the CSPAD, defining explicit readout directions (**d**_*x*_ and **d**_*y*_) accounts correctly for the pinwheel construction of the device, where quadrants have an approximately 90° relation to each other, rotated around a common origin. Within each quadrant, groups of two sensors also have an approximate 90° relationship (Fig. 1 ▸). Therefore, in contrast to the monolithic array of Ha14, the present design implements separate data arrays corresponding to each sensor, which, while having a common layout in memory, represent four distinct orientations in space.

To implement our replacement for Ha14, we adopted the *DIALS* software framework (*Diffraction Integration for Advanced Light Sources*; Winter *et al.*, 2018 ▸) that has previously been used for inverse modeling of synchrotron-based rotation crystallography experiments (Waterman *et al.*, 2016 ▸). One relevant difference is that while rotation experiments generally treat one crystal at a time, the refinement of multipanel detector geometry required us to combine Bragg positional data from thousands of crystals. The parameter-fitting problem is thus highly interdependent, with all detector-panel positions feeding into the refinement of the orientation and unit-cell parameters of each crystal, while simultaneously each crystal model determines the positions of all of the detector panels. Standard methods in iterative least-squares parameter refinement, such as the Levenberg–Marquardt algorithm (§[Sec sec4]4), involve the construction of a set of linear equations with as many unknowns *n* as free parameters; therefore, an *n* × *n* normal matrix must be decomposed (Bevington & Robinson, 2003 ▸). Naively expressed, this is a very large matrix; for example, 32 sensor tiles with *xy* translations and one rotation each, plus 3000 hexagonal crystals with three orientation angles plus *a* and *c* parameters, would produce a total of *n* = 15 096. As a short cut, the work presented in Ha14 employed alternating cycles of refinement, alternating between the detector panels and the individual crystal models, such that the full matrix is never constructed. However, for the work presented below, we wished as a general principle to minimize the construction of arbitrary refinement pathways (such as detector panels first then crystal models) and to rely whenever possible on the global refinement of all free parameters. To this end, we exploited the fact that many of the parameters are independent (for example, all of the cross-terms involving two distinct detector panels or two distinct crystals contribute zero-valued coefficients to the normal equations). Since the sparsely dependent structure of the normal equations is known ahead of time, we show (§[Sec sec4]4) how sparse linear algebra techniques can be employed to substantially reduce the computational resources needed to solve the problem.

Also, we show below how the *DIALS* framework can be adapted to describe serial crystallography experiments involving two imaging detectors at different crystal-to-detector distances (§[Sec sec5]5) and how the simultaneous refinement of detector and crystal models improves the accuracy of poorly measured unit-cell axis lengths for unit-cell axes that are oriented nearly parallel to the X-ray beam (§[Sec sec6]6). Finally, considering recent reports from other groups describing how small changes in the crystal-to-detector distance can affect experimental results (Nass *et al.*, 2016 ▸), we develop a procedure to discover small time-dependent changes in the distance, thereby improving the integrated Bragg spot signal (§[Sec sec7]7).

## 2. Data sets   

We reprocessed thermolysin diffraction patterns collected at the CXI endstation at LCLS (Table 1 ▸). Sample preparation and injection, beamline parameters and data-collection methods are described in Kern *et al.* (2014 ▸). 760 110 shots were collected over 107 min, with an incident photon energy set point of about 9.75 keV. The front CSPAD was positioned at either 130 or 105 mm from the sample-insertion position, while the back detector was positioned ∼2.5 m from the sample-insertion position, so that low-angle diffraction transmitted through the central aperture of the front CSPAD was collected on the back detector. Data collection occurs in ‘runs’, which represent continuous time intervals (typically 5–10 min each) during which experimental parameters are held constant. The runs listed in Table 1 ▸ are grouped by front detector distance.

We also reprocessed Cry3A toxin data collected about a week later on the same CSPAD detector at CXI. 380 740 shots were collected over 53 min at an incident photon energy set point of 8.5 keV. Sample preparation and injection, beamline parameters and data-collection methods are described in Sawaya *et al.* (2014 ▸).

## 3. CSPAD detector metrology refinement   

We refined the CSPAD detector metrology using custom-written code for serial crystallography incorporated into *dials.refine*. §[Sec sec3.1]3.1 describes the hierarchical organization of the CSPAD and §[Sec sec3.2]3.2 describes the automatic determination of initial quadrant locations using powder patterns. Given this initial alignment, we can index the data (§[Sec sec3.3]3.3), perform joint refinement on the detector and crystal models (§§[Sec sec3.4]3.4 and [Sec sec3.5]3.5) and assess the accuracy of the results (§[Sec sec3.6]3.6).

### 3.1. CSPAD hierarchy   

Our detector model represents the panels of the CSPAD in a four-level hierarchy (Fig. 1 ▸): detector, quadrant, sensor and ASIC. Switching the local frame of reference between levels involves a coordinate transformation **F**_parent→child_, defined as a change of basis from a parent coordinate system to a child coordinate system or back again (*i.e.* **F** ^−1^_parent→child_ = **F**_child→parent_). The transformation **F** can be expressed with an origin vector that translates from the origin of the parent frame to the origin of the child frame and a unitary matrix describing a rotation. The first frame shift, **F**_lab→d_, moves from the laboratory origin (*i.e.* the crystal location) to the center of the detector as a whole. Next, we describe four detector-to-quadrant frame shifts, **F**_d→q0_ through **F**_d→q3_. There are then 32 quadrant-to-sensor frame shifts, **F**_q*i*→s0_ through **F**_q*i*→s7_, where *i* ranges from 0 to 3. Finally, since all pairs of ASICs are constrained to have the same three-pixel gap between them, there are exactly two sensor-to-ASIC frame shifts, **F**_s→a0_ and **F**_s→a1_, applied to all ASICs.

The full transformation of position **p** from the laboratory frame to the ASIC frame would be expressed as
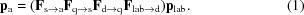
For convenience, we express **F**_lab→d_ as a homogenous transformation matrix consisting of the components of the rotation matrix (**d**_*x*_, **d**_*y*_, **d**_*n*_) (where **d**_*n*_ is the normal vector, **d**_*x*_ × **d**_*y*_), and the translation vector **d**_0_:
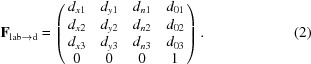



The other frame shifts are expressed in the same form but are generated from different sets of **d**_0_, **d**_*x*_ and **d**_*y*_ vectors. The 4 × 4 homogenous transformation matrix allows both rotation and translation to be expressed by single matrix multiplication. The global, cumulative **d**_0_, **d**_*x*_ and **d**_*y*_ vectors used to compute pixel positions (Parkhurst *et al.*, 2014 ▸) can then be easily derived from (1) given a cumulative frame shift **F**:
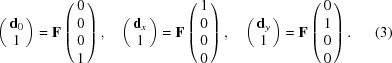



In (3), after multiplying **F** by a four-element vector, we drop the last element in the resultant homogenous vector to construct the **d** vectors. The location of a pixel in the plane of the ASIC chip is determined using a pixel-to-millimetre conversion function that takes into account pixel size (including rectangular pixels and optionally parallax effects; Parkhurst *et al.*, 2014 ▸; Waterman *et al.*, 2016 ▸).^1^

The reverse operation (determining **p**_lab_ given **p**_a_) can be performed using reversed and inverted matrix multiplications from (1):
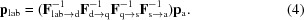



These transformations group the 64 ASICs into hierarchical sets. With this organization, it is possible to refine the detector as a whole without modifying the frames of child components or to refine a quadrant as a whole without modifying the frames of its parent or children, and so on. Detailed specifications as to how these transformations are recorded have been presented previously (Brewster *et al.*, 2014 ▸).

### 3.2. Automatic CSPAD quadrant alignment using rotational autocorrelation   

Within each CSPAD quadrant, the initial panel positions are determined at the LCLS facility using an optical microscope. Therefore, when the CSPAD is assembled and installed, the positions of the quadrants relative to each other and to the beam are unknown.

**Figure 2:**
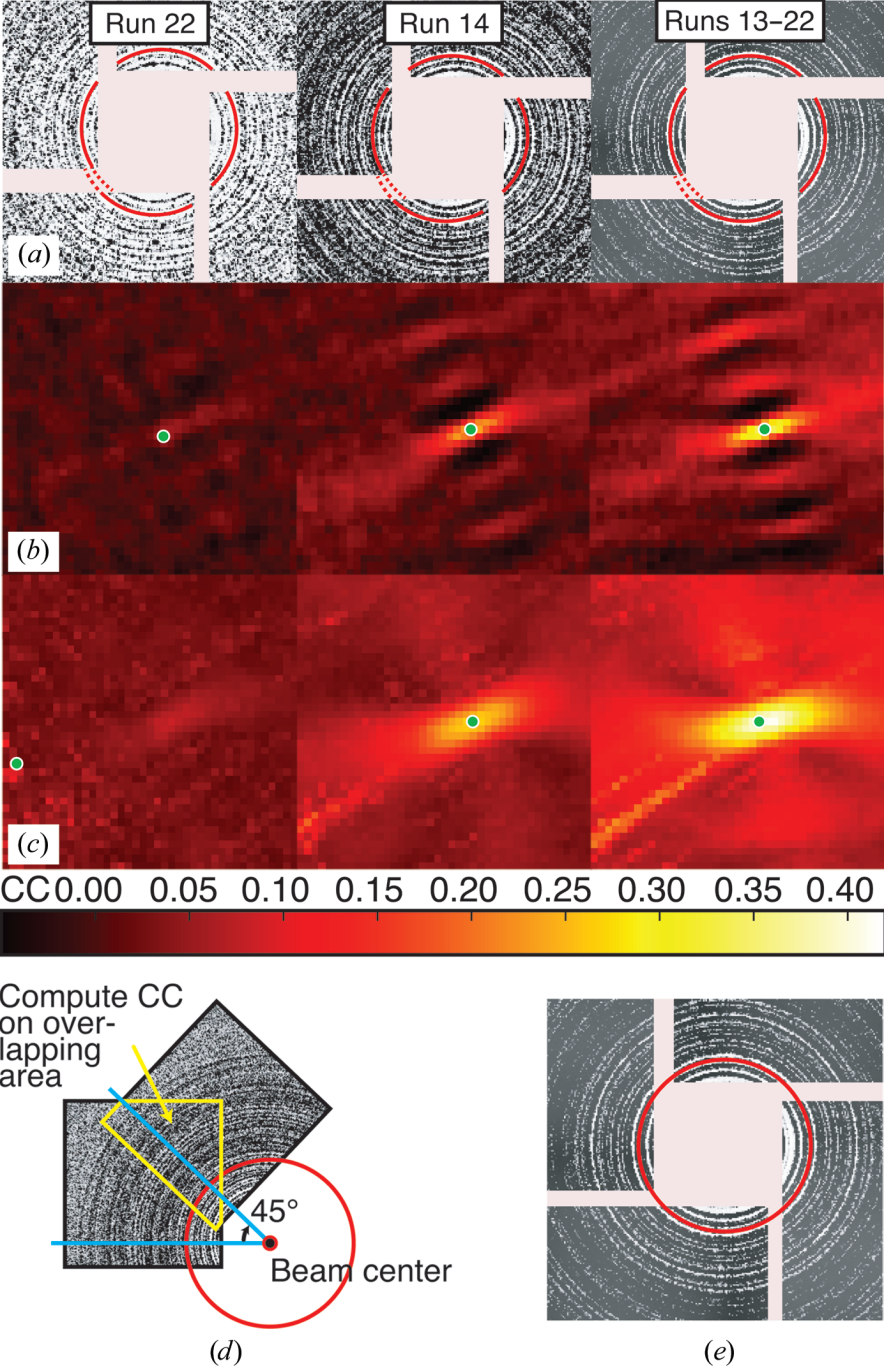
Automatic quadrant alignment by rotational autocorrelation from thermolysin data. (*a*) Composite maximum images from run 22, run 14 and runs 13–22. The red arcs and dotted extensions have been placed to show that the virtual powder rings are not circular. (*b*) Rotational autocorrelations of quadrant 2. The *x* and *y* axes represent the incremental shifts in the quadrant position used to calculate the autocorrelation, with the center coordinate (0, 0) representing no shift. The heat map is colored by the rotational autocorrelation of the quadrant with itself, rotated 45° around the beam center. Heat maps are all colored on the same scale (see color bar). The maximum value is marked with a green dot. (*c*) Autocorrelation map using multiple rotation angles. Each point is the maximum CC value found when rotating the panel in steps from 20 to 70° in 2.5° increments. (*d*) Diagram illustrating rotational autocorrelation. The quadrant is rotated 45° and a CC is computed between the pixel values in the overlapping areas. This is repeated for each of the grid points after translating the quadrant. (*e*) The maximum composite pattern from runs 13–22 after applying the *x* and *y* shifts for each quadrant that maximize the rotational autocorrelation.

We developed an automated approach for deriving quadrant positions; more specifically, we calculate the *xy* positions (in the detector plane) of the four sensors closest to the direct beam (Fig. 1 ▸, shaded in pink). Firstly, we generate a ‘composite maximum’ image, taking the maximum pixel values among all images in a data-set run. Overlaying all of the Bragg spots from a run in this manner generates a virtual powder pattern, since the individual crystals have random orientations. If a quadrant is properly placed, after rotating a strong powder pattern 45° around the beam center, the overlapping pixel values will be highly correlated. We can therefore perform a grid search over *xy* offsets for each quadrant separately (limiting our examination to the sensor closest to the beam center), searching for the position with the highest rotational autocorrelation coefficient (CC). This produces a heat map (Fig. 2 ▸) where each pixel [for example (3, 4)] represents the CC when the quadrant is translated by that amount (three pixels in *x*, four pixels in *y*). The coordinates of the heat-map maximum give the best positional correction for that quadrant (Table 2 ▸).

We tested three virtual powder patterns to estimate the quadrant positions for the front CSPAD detector using the thermolysin data (Fig. 2 ▸ *a*). The first is derived from a weak run (run 22) with few hits, leading to thin powder rings. The second is derived from a strong run (run 14) with many hits. The third is a composite of many runs collected at a single detector distance (runs 13–22). In all three patterns, the discontinuities in the powder rings indicate that the quadrants are not aligned.

Fig. 2 ▸(*b*) shows the rotational autocorrelation heat maps for these three virtual powder patterns. For the weakest, run 22, the maximum of the heat map is unclear. It is better resolved for run 14 and is strongest for the composite pattern (runs 13–22). Alternating bands of low and high correlation occur owing to the alternating strong and weak bands in the powder pattern. When, after translating the quadrant, bands in the 45° rotation overlap with similarly bright bands in the unrotated pattern, the CC is higher. Likewise, the translation can cause bands in the rotated pattern to systematically overlap gaps in the unrotated pattern and produce a low CC.

For weaker patterns or sparser data, it can be useful to try many rotations. We repeated the rotation test for each of the virtual powder patterns at different angles from 20 to 70° in 2.5° increments. For each *xy* offset, we selected the maximum CC observed from all of the angles tested to generate a new heat map. This eliminates the ‘beat pattern’ of local maxima in single-angle heat maps and in general produces smoother heat maps with clear global maxima (Fig. 2 ▸ *c*). For weak data (for example run 22), however, the global maximum may still be very far off from the best quadrant position. In such cases, it is prudent to cross-check by manual examination of virtual powder patterns overlaid with circles emanating from the beam center.

Once the quadrant positions have been optimized, it is often possible to estimate the sample-to-detector distance to within ∼1 mm (data not shown) by overlaying the corrected composite image with predicted rings calculated from the known unit-cell parameters of the protein producing the virtual powder pattern.

### 3.3. Initial indexing   

After refinement of the quadrant positions by rotational autocorrelation, we performed initial indexing of the thermolysin data on the front CSPAD to generate a data set from which we could refine the complete detector metrology. We determined a starting detector distance by performing rounds of indexing at increasing distances and choosing the distance producing the highest number of indexed images, as described previously (Hattne *et al.*, 2014 ▸). We then indexed all of the images, determining initial basis vectors using the one-dimensional Fourier method (Steller *et al.*, 1997 ▸), while guiding the selection of candidate basis vectors using a target unit cell (*a *= *b* = 92.9, *c* = 130.4 Å, α = β = 90, γ = 120°; Hattne *et al.*, 2014 ▸). For each image, we independently refined the crystal orientation matrix and unit-cell parameters, while holding the wavelength and detector position constant. This produced 118 318 indexed lattices from both detector distances (∼130 and 105 mm). In subsequent sections, we refine the detector metrology using a subset of these lattices from initial indexing (§§[Sec sec3.5]3.5 and [Sec sec3.6]3.6) and then use the refined metrology to reindex the data, producing successfully indexed patterns that previously did not index (§[Sec sec3.7]3.7).

### 3.4. Refinement target function   

Computational models for still-shot experiments were incorporated into the parameter-refinement framework (Waterman *et al.*, 2016 ▸) within *DIALS*. Best-fit models for the detector, crystal and beam were determined by minimizing the nonlinear least-squares target function
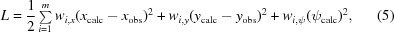
where the index *i* traverses all *m* Bragg spot measurements in the entire data set, *x* and *y* refer to the fast and slow coordinates of the Bragg spot centers of mass on their respective detector panels, the subscript ‘obs’ refers to the observed position and the subscript ‘calc’ refers to the position predicted by the computational model. The quantity ψ_calc_ is the smallest crystal rotation angle required to place the reciprocal-lattice point exactly in the reflecting condition described by Bragg’s law. As described previously (Sauter *et al.*, 2014 ▸), it is necessary to include this restraint to prevent the crystal orientation model from rotating around axes that are perpendicular to the beam vector, as these rotations do not directly change the Bragg spot positions. The weighting scheme (*w*_*i*,*x*_, *w*_*i*,*y*_ and *w*_*i*,ψ_ for the *i*th observation) uses a statistical weight for *w*_*i*,*x*_ and *w*_*i*,*y*_ equal to the inverse variance of the observed spot position and a constant weight for the ψ_calc_ angle. The default *w*_*i*,ψ_ value of 10^6^ generally puts the ψ_calc_ term on the same scale as the *x* and *y* terms.

### 3.5. Refinement of the detector model   

To determine the correct positions of the CSPAD detector panels to subpixel accuracy, we conducted iterative nonlinear least-squares parameter optimizations designed to jointly refine the detector geometry and crystal models. We used Bragg spot positions measured on thermolysin diffraction images selected from the 130 mm run group (Table 1 ▸) and limited our refinement to the 3000 images with the most reflections indexed to the corners of the detector. In addition to the geometric degrees of freedom of the detector, explained below, we treated the two hexagonal unit-cell lengths and the three crystal orientation angles as free parameters refined independently for each shot. The beam direction is considered to be static, and since each X-ray pulse has slightly different mean energy, measured by the beamline instrumentation, this measured energy is used here without refinement.

**Figure 3:**
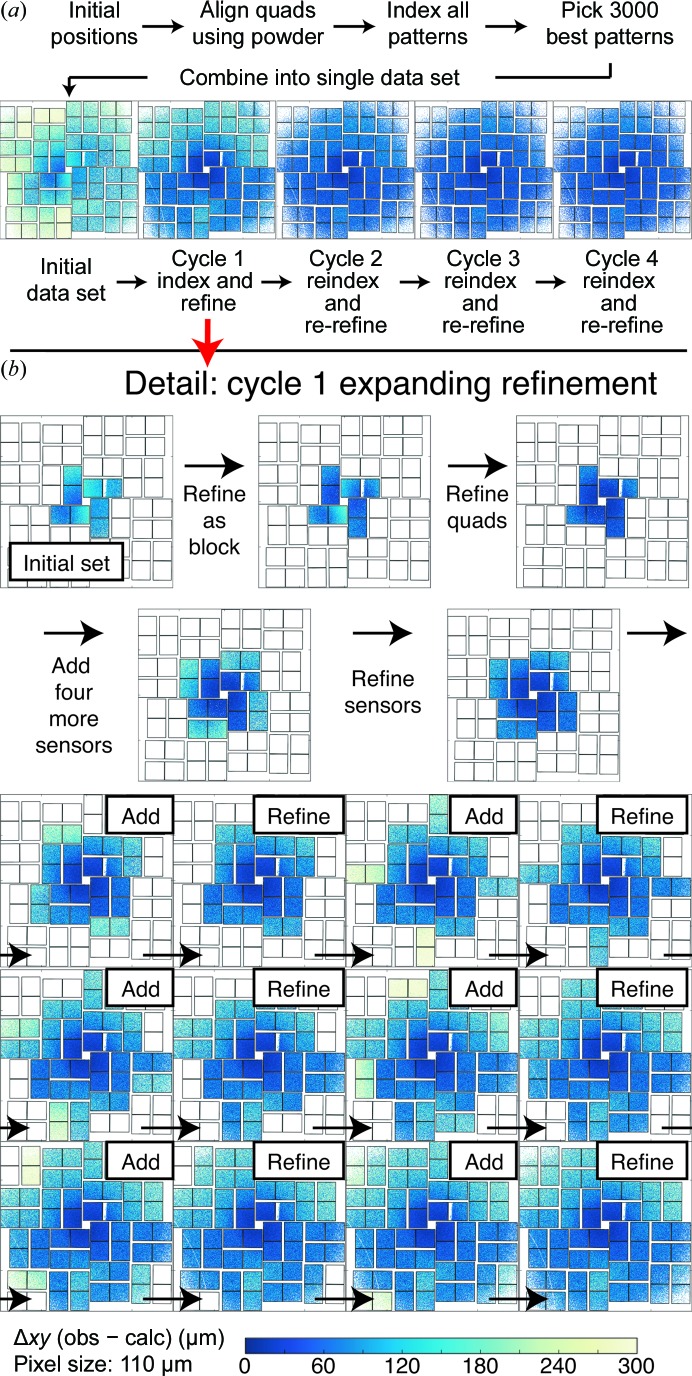
Iterative CSPAD refinement. (*a*) Using initial positions provided by optical measurement of the panel positions, the quadrants are aligned using a powder pattern and all patterns are then indexed. The best 3000 images are combined into an initial data set (leftmost image). The layout of the CSPAD is shown with every indexed reflection plotted as a single dot. The dots are colored by Δ*xy*, the magnitude of the difference between the observed (obs) and predicted (calc) spot locations [see the color bar at the bottom of (*b*); blue indicates a prediction nearly matching the observation, while green through yellow indicate poorly predicted reflections]. Cycle 1 shows Δ*xy* after the first round of refinement. Subsequent cycles (2–4) show iterations of reindexing using the new metrology and re-refinement of that metrology. (*b*) Detail of cycle 1 in the expanding-mode refinement. After the initial set of reflections is selected on the inner four sensors, the detector is refined as a group and the quadrants are then refined separately. The next four sensors out are then added and the eight sensors are refined independently. This continues until the entire detector is refined.

Two different protocols were developed, ‘hierarchical mode’ (Table 3 ▸) and ‘expanding mode’ (Table 4 ▸), consisting of sequences of either three or nine optimizations, respectively, with each step in the sequence including a wider list of free geometric parameters describing the detector. The general motivation for this was to avoid trapping the detector geometry in a local minimum of (5) and instead to refine the most reliable parameters first. In particular, the ‘expanding mode’ protocol refines the positions of the four sensors (one per quadrant) closest to the direct beam, before sequentially adding groups of sensors at larger diffraction angles, as illustrated in Fig. 3 ▸.

The refinable parameters for a detector panel or group of panels include distance (the translation along **d**_*n*_), Shift1 and Shift2 (the translations along **d**_*x*_ and **d**_*y*_) and τ_1_, τ_2_ and τ_3_ (the rotations around **d**_*n*_, **d**_*x*_ and **d**_*y*_, respectively). Tables 3 ▸ and 4 ▸, which list the details of the two protocols, summarize which geometric parameters are refined as the optimizations progress from the entire detector to each quadrant separately and finally to individual sensors. Each row represents a separate refinement operation of up to 3000 crystal models and one detector model. The output models from each row are used as inputs for the next. Since the crystal rotation around the beam axis is directly correlated with the detector rotation around this axis, we fix τ_1_. This is different from rotation crystallography as rotation around the goniometer breaks the degeneracy between the detector and crystal rotations around the beam axis. However, we do refine τ_1_ at the levels of the individual quadrants and sensors. Furthermore, we fixed the detector τ_2_ and τ_3_. This is not strictly necessary, as *DIALS* is capable of refining three translations and rotations for all detector elements. Refinement of the tilt is routinely performed for synchrotron data. However, for this particular experiment we found that refinement of the detector τ_2_ and τ_3_ produced little difference in the results (data not shown). For the quadrants and sensors we fixed τ_2_ and τ_3_ as well as distance offsets, so as to constrain all detector panels to be coplanar, because we considered the refinement of independent panel tilts and distances to be beyond the scope of this study.

As detailed in Tables 3 ▸ and 4 ▸, our sequence of optimizations begins at the level of refining the overall detector distance and *xy* shift and ends at the level of individual sensor *xy* shifts and τ_1_ rotations: we do not refine the relative positions of the 2 × 1 pairs of ASICs independently. Doing so would have no physical meaning, as each pair of ASICs is bonded to a single chip, oriented by lithography and uniformly three pixels apart from one another, and any deviations from this are understood to be the result of absorbing error elsewhere in the model. Finally, when refining the τ_1_ angles at the level of individual quadrants or sensors, we are careful to lock down one of them (the first one in each group, τ_1group1_), since only *N* − 1 of the angles are independent, where *N* is the number of detector panels to be refined. In other words, in Table 3 ▸ one of the four quadrants in row 2 is fixed and one of the 32 sensors in row 3 is fixed.

After the entire optimization protocol has ended, we repeat the entire cycle up to four times to assess the ability to converge to a stationary solution (Fig. 3 ▸ *a* and Tables 5 ▸ and 6 ▸). We defined convergence as (i) the root-mean-squared differences (r.m.s.d.s) between observed and calculated spot positions no longer decreasing and (ii) the detector positions no longer shifting appreciably. Before each subsequent cycle, the 3000 images were reindexed and crystals with a poor r.m.s.d. were discarded as outliers (Brewster *et al.*, 2016 ▸). For this reason, subsequent cycles have fewer than 3000 crystals contributing to the joint refinement (Table 5 ▸).

### 3.6. Refinement accuracy and precision   

The greatest improvement in r.m.s.d. is found after cycle 1 (Fig. 3 ▸ *a* and Table 5 ▸, expanding protocol), but reindexing the images and re-refining gives an additional improvement (cycle 2). It is expected that the metrology should converge rapidly, and we see that subsequent cycles do not appreciably improve the spot predictions, so we regard cycles 3 and 4 as controls.

Again, each cycle (1–4) consists of indexing and refinement. The initial data set consists of ∼700 000 reflections across 3000 images with an r.m.s.d. of 221 µm. During refinement, outlier rejection (Sauter & Poon, 2010 ▸) reduces this data set to ∼580 000 reflections. After reindexing and re-refinement (which again includes outlier rejection), cycle 2 produces a data set with only ∼330 000 indexed reflections. In this work, we do not investigate the cause of this fall-off; however, we note that it is associated with a radial streaking of reflections at high resolution (Hattne *et al.*, 2014 ▸) producing poorly measured spot centroids for reflections with a high Δψ angle. Refinement moves the tiles such that these reflections are no longer close enough to their predictions to be indexed with our monochromatic beam model, since when the centroids from the elongated reflection are moved to reciprocal space their non-integer Miller indices are no longer close enough to a round number (the default cutoff is 0.3 of a whole integer). Because of this, and because r.m.s.d. is sensitive to sample size, we additionally computed an r.m.s.d. using only those reflections indexed in all cycles and both modes. This ‘common set’ of reflections, as determined by observed spot centroids in pixels (an invariant property of the reflections), consists of 218 954 reflections. Determining the r.m.s.d. on only this set ensures that the cycles and modes are comparable. We can see that most of the improvement occurs during a single round of indexing and refinement (cycle 1, 157.9 to 60.1 µm in the expanding case, or roughly half a pixel for these 110 µm CSPAD pixels). An additional round of reindexing and refinement (cycle 2) improves the expanding case by a small amount to 50.1 µm. Cycles 3 and 4 do not appreciably change the r.m.s.d. or tile positions (Table 6 ▸). Note that the average change in sensor position (Δ*xy*) is 9.8, 11.1 and 9.0 µm in cycles 2, 3 and 4, respectively, while the overall detector and quadrant shifts are much lower (<2 µm). This suggests that reindexing and reoptimization impart random *xy* shifts at the sensor level while not appreciably improving the accuracy, thus giving a rough estimate for the precision of about 10 µm. This is similar to the precision that we previously reported for the Ha14 method.

An interesting trend can be seen in cycles 3 and 4, in which minimal improvement in r.m.s.d. after refinement is seen. Hierarchy level 0, the whole detector, is refined using only the innermost reflections. During cycles 3 and 4 the detector as a whole shifts away from the crystal by nearly 100 µm. Then, after all the sensors have been added while refining at hierarchy level 2, the detector distance shifts back to where it started. This indicates a small, resolution-dependent mis­prediction of reflections.

### 3.7. Reindexing using refined metrology   

With cycle 4 of the expanding refinement showing the best r.m.s.d.s, we repeated the indexing and integration of all shots with this metrology, including searching for a second lattice from a possible second crystal on each image. We also took into account the small change in detector distance from the final refined model (a decrease of about 36 µm), and we used as a target unit cell the mean unit cell of the refined crystal models (*a* = *b* = 93.28, *c* = 130.81 Å). This produced 119 774 primary lattices (an improvement of nearly 1500 lattices from the unrefined metrology) and 46 476 secondary lattices, giving a total of 166 250 lattices.

## 4. Refinement engines and sparse matrices   

Parameter-optimization engines require a target function to be minimized, a set of parameters and a set of observations. A given engine will modify the parameters in ‘steps’ and accept the incremental change if the target function decreases. The direction of the step is generally determined by a gradient vector consisting of the first derivative ∂*L*/∂*p* of the target function *L* with respect to each parameter *p*, which indicates how each parameter needs to change to lower the target function. The parameters that have the most influence on the target function can be determined using curvatures, the second derivatives ∂^2^ *L*/∂*p* ^2^ of the target with respect to each parameter, which determine (in an inverse sense) the step size for each parameter. The engine continues to take steps until some convergence criteria are reached. The overall scale of our optimization problem is uncommonly large, with tens or hundreds of thousands of free parameters (§[Sec sec1]1), and as such is not adequately treated in recent macromolecular crystallo­graphy diffraction modeling literature. We therefore survey the available methods briefly, investigating three potential approaches in common crystallographic use, before finally choosing a fourth method that is adopted from the sparse-algebra community.

Firstly, the limited-memory Broyden–Fletcher–Goldfarb–Shanno (LBFGS) algorithm is a quasi-Newton method that uses a low-memory approximation to the second-derivative matrix (the Hessian matrix) to compute a step size between iterations, and is thus suitable for optimization problems with large number of parameters (Liu & Nocedal, 1989 ▸). It does not rely on analytical second derivatives that can be difficult to derive and thus present a barrier to programming. However, with a large number of parameters LBFGS has a poor convergence rate, as hundreds to thousands of steps may be necessary to find a minimum.

Construction of the approximate Hessian may be seeded by providing a vector of curvatures representing the diagonal elements of the Hessian matrix. Providing the curvatures can dramatically improve the performance of LBFGS, and thus constitutes our second optimization method. As implemented in *DIALS*, the second partial derivatives are not analytically calculated.^2^ However, we can use the assumption that the target function *L* is of least-squares form to calculate an approximate value for the second derivative,
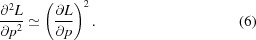
This is the approximation used in the Gauss–Newton algorithm as a modification of Newton’s method (see equation 10.24 in §10.3 of Nocedal & Wright, 2006 ▸). The approximation has the pleasing property of improving as the refinement approaches convergence. In many instances, we find that supplementing the LBFGS algorithm with approximate curvatures is essential for reducing the number of steps before convergence to an acceptable level.

**Figure 4:**
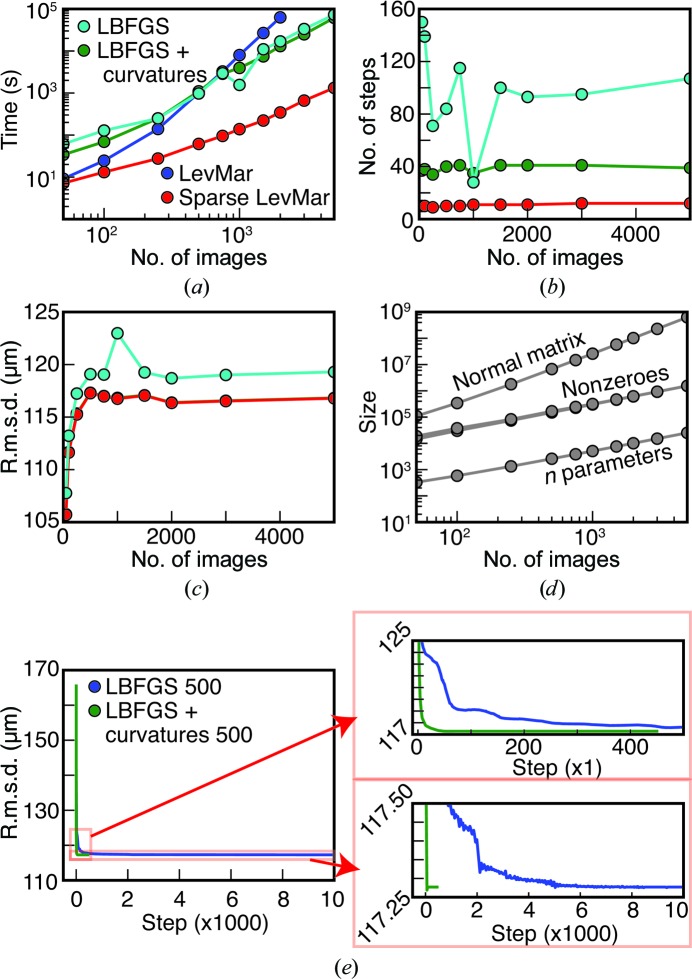
Comparison of four different refinement engines: LBFGS, LBFGS with curvatures, LevMar and sparse LevMar. 50–5000 crystal models and each of the 32 sensors were refined simultaneously. (*a*) Total run time for refinement, averaged over ten trials. (*b*) Number of steps taken by each engine during refinement (LevMar and sparse LevMar exactly overlap). (*c*) Data-set r.m.s.d.s (obs − calc) from each refinement engine. Except for LBFGS, the traces from the engines overlap. (*d*) Array sizes for Levenberg–Marquardt. A normal matrix used for refining *n* parameters contains *n*(*n* + 1)/2 elements in the upper triangle, of which only a subset are nonzero. Therefore, the number of elements in the normal matrix grows faster than *n* and faster than the number of nonzero elements in the normal matrix. (*e*) Extended refinement of 500 images using LBFGS and LBFGS with curvatures. 10 000 steps are shown after removing the r.m.s.d. convergence check during refinement. R.m.s.d. *versus* step number is shown on the left. Two enlargements are provided on the right. Top enlargement: early refinement steps. Bottom enlargement: all refinement steps but enlarged tightly in r.m.s.d. to show small changes over time.

LBFGS, even with curvatures, can still take too many steps for our joint refinement of the detector and crystals. We previously noted (Waterman *et al.*, 2016 ▸) that a third algorithm, based on Gauss–Newton methods, but modified by the Levenberg–Marquardt (LevMar) approach to remain robust in the presence of covariance, would be ideal since it explicitly takes account of the nonlinear least-squares form of the target function and as a result requires far fewer steps, while avoiding any need for second derivatives. Briefly, the Gauss–Newton method derives the vector **δ** of increments to the current parameter estimates by utilizing the Jacobian matrix **J**, defined as the matrix of partial derivatives of each residual term *r* with respect to each parameter. In this matrix, row *i* represents the derivative of the *i*th residual. Note that there are *m* observations but 3*m* residuals, since (5) gives three residuals (*x*_calc_ − *x*_obs_, *y*_calc_ − *y*_obs_ and ψ_calc_) for each measurement. Furthermore, column *j* represents the derivative with respect to the *j*th freely refined parameter, with *n* total parameters:
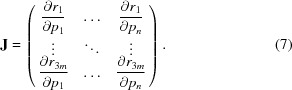



Matrix **J** is used to construct a set of normal equations **Aδ** = **b**, where **A** = **J** ^T^ **J** and is a symmetric matrix of size *n* × *n*, **b** = −**J** ^T^ **r** and **r** is the vector of all residuals (Nocedal & Wright, 2006 ▸). LevMar modifies **A**, adding a damping factor that affects the step size (Bevington & Robinson, 2003 ▸; Nocedal & Wright, 2006 ▸). Considering the large matrix sizes here, it is prohibitively expensive to explicitly compute the inverse matrix **A** ^−1^ in order to solve for **δ**. However, it is possible to perform a Cholesky decomposition, expressing the matrix as a product of a lower triangular matrix **L** and its transpose, **A** = **L** ^T^ **L**, and then to derive **δ** by back-substitution. While this algorithm offers the best convergence behavior for optimization sizes typical of rotation-based crystallography (Waterman *et al.*, 2016 ▸), the performance of the Cholesky decomposition limits problem sizes to *n* < 5000 (Fig. 4 ▸ *a*).

To overcome this performance limitation, we took advantage of the fact that both the Jacobian and normal matrices for our problem are sparse, meaning we have prior knowledge that most of the elements are zero. Elements of the normal matrix are (structurally) zero when they represent cross-terms between independent free parameters. For example, the unit-cell parameters and orientation angles for a given crystal are independent of the parameters describing all of the other crystals. Only the cross-terms relating detector-panel parameters and crystals are in general nonzero. Knowing which elements of **A** are zero leads us to deduce, using graph theory, which elements of the Cholesky factor are also structural zeroes (Liu, 1990 ▸; Mehta & Sahni, 2004 ▸; Rennich *et al.*, 2014 ▸), thus allowing a dramatically reduced level of computational effort to calculate the nonzero elements of the Cholesky factor. Sparse-matrix Cholesky decomposition methods, which are well known in mathematics but not in crystallography, therefore afford us our fourth algorithmic approach, and the only one that is suitable for a 3000-crystal problem. We incorporated the open-source Eigen linear-algebra library (Guennebaud & Jacob, 2010 ▸) into our software distribution for this application.

To evaluate the performance and memory requirements of the four engines, we simultaneously refined between 50 and 5000 random images from a single run jointly with the 32 sensor positions (Fig. 4 ▸). For each problem size and each engine, we performed ten independent trials to obtain an average run time, accounting for local variation in the computing environment. Time trials were run single-process on a 12-core, 64-bit Intel Xeon X5675 processor (3.07 GHz) with a 12 MB cache and 24 GB RAM running Red Hat Enterprise Linux Server 7.3. C++ code was compiled under GCC 4.8.5. The initial detector model had quadrants aligned using rotational autocorrelation from a powder pattern, but was otherwise unrefined, meaning that this refinement was equivalent to the third step of Table 3 ▸, skipping the first two steps. Starting with the largest data set, each smaller data set was a random subset of the next largest. We disabled outlier rejection in order to focus on the performance of the refinement steps themselves. We averaged the ten run times, but since the inputs were the same the r.m.s.d.s, number of steps until convergence and memory usages (Figs. 4 ▸ *b*, 4 ▸ *c* and 4 ▸ *d*) were constant within each group of ten trials. Sparse-matrix LevMar had the shortest run time of the four (Fig. 4 ▸ *a*), while the LBFGS implementations were markedly slower owing to the number of steps that were needed for convergence. Once the number of parameters exceeded 5000, LevMar became completely unacceptable in its run time. Indeed, the final data points using 3000 and 5000 images were terminated after 48 h in the queue at LCLS. Of the four, LBFGS without curvatures took the most steps to converge (Fig. 4 ▸ *b*) and had slightly poorer r.m.s.d. values than the other three (Fig. 4 ▸ *c*; see below).

Fig. 4 ▸(*d*) shows the memory savings achieved using sparse-matrix LevMar compared with LevMar. The normal matrix size (the square of the number of parameters *n*) is shown on a log scale. Likewise, the number of nonzero values in the normal matrix and the number of nonzero values in the Cholesky factor are shown (these nearly overlap). The size of the normal matrix increases on the order of *k* ^1.89^, where *k* is the number of images, while the number of nonzero values increases on the order of *k* ^0.95^ to *k* ^1.01^ (Table 7 ▸). The savings in memory by using sparse matrices is concurrent with a decrease in run time as a function of the number of images, from order *k* ^2.45^ for LevMar to *k* ^1.13^ for sparse-matrix LevMar.

To clearly show the convergence behavior of LBFGS, we removed the termination condition where refinement stops if the r.m.s.d.s cease changing within a certain threshold and ran the refinements for 10 000 steps (Fig. 4 ▸ *e*). LBFGS with curvatures still terminated early owing to round-off checks in the LBFGS minimizer, but the same final r.m.s.d. was reached by both LBFGS engines. LBFGS took over 6000 steps to reach the r.m.s.d. that LBFGS with curvatures reached in less than 500 steps.

## 5. Advanced refinement: a second detector   

**Figure 5:**
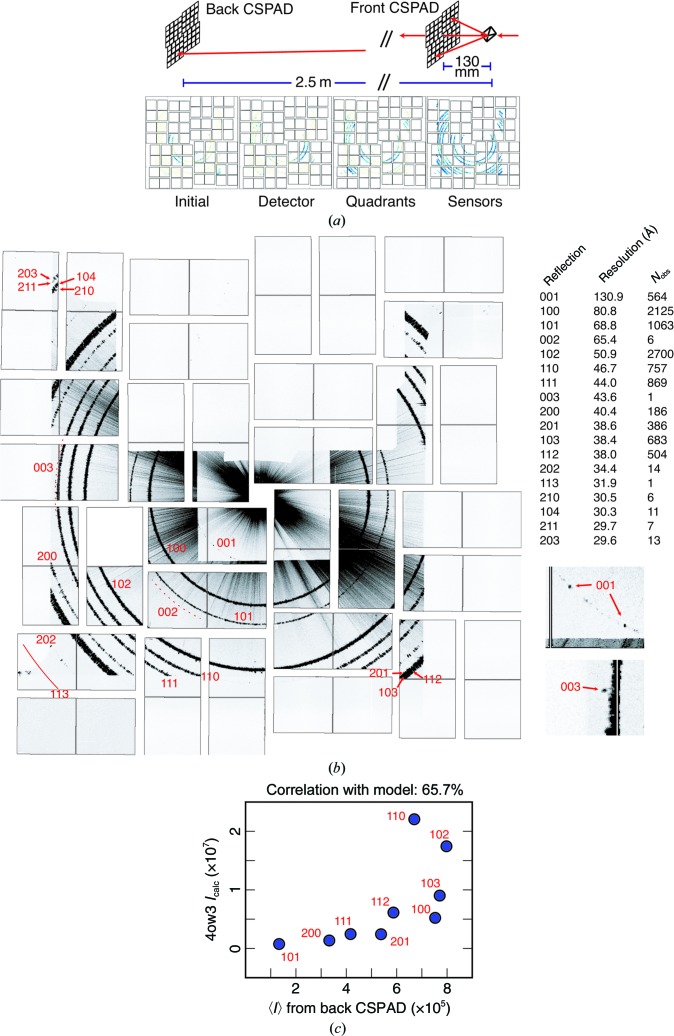
Back detector refinement. (*a*) Top: experimental setup. High-angle diffracted X-rays are recorded on the front CSPAD, while low-angle Bragg reflections are recorded on the back detector, approximately 2.5 m from the crystal. Bottom: Δ*xy* plots for hierarchical mode refinement of the back detector, showing Δ*xy* initially and after refining the detector, quadrants and sensors, colored as in Fig. 3 ▸. Regions with no diffraction were shadowed by equipment in the beam path. (*b*) Maximum composite of runs 11–22, showing refined sensor positions. Rays extending radially from the beam center arise from diffraction from the liquid jet. Rings are numbered according to their Miller indices. Rings 001, 002 and 003 are systematic absences in space group *P*6_1_22; 564 reflections were indexed for 001, but for 002 and 003 fewer than ten each were found (dashed lines). Only one reflection from 113 was observed (solid red line). Enlarged views of 001 and 003 are shown on the lower right. Reflections are listed in a table on the right along with their resolution and the multiplicity of observation. (*c*) Correlation of calculated intensities from PDB entry 4ow3 with average intensities from reflections indexed on the back CSPAD. Intensities from the back CSPAD are unscaled and otherwise uncorrected. Nonsystematically absent reflections with at least 20 observations are shown, and they have a CC of 65.7% with the intensities calculated from the reference structure.

The *DIALS* refinement platform is highly flexible and configurable, being capable of refining experimental geometry from many crystals and detectors, even if the diffraction pattern is spread across more than one detector simultaneously. As an example, we refined the metrology of the back CSPAD using thermolysin patterns (Table 1 ▸) recorded on both the front and back detectors. The front detector, located either 130 or 105 mm from the crystal position, has a central aperture that, while designed to transmit the nondiffracting beam, also permits low-angle Bragg reflections to be recorded on the back detector approximately 2.5 m from the sample (Fig. 5 ▸ *a*, top). A detector in this position is not typically used in XFEL experiments, but we can cite two possible roles. Firstly, one can record the fine detail of low-resolution reflections. As the large crystal-to-detector distance spreads the reflections out over many pixels, we can potentially analyse the spot shape and mosaic character of the crystals. Secondly, we used the back CSPAD in Duyvesteyn *et al.* (2018 ▸) to examine bacteriophage phiX174, a crystalline viral condensate with a large unit cell (∼500 Å) that diffracted to poor resolution (∼50 Å), to the point where diffraction was only seen on the back detector. An analysis of the Bragg spots revealed the space group and approximate unit-cell dimensions, given a histogram of the reciprocal-space reflection distances. Both these use cases can benefit from an accurate detector metrology; therefore, with thermolysin as a standard, we used crystal orientations from lattices recorded on the front detector to index reflections on the back detector from the same crystal and then refined the back detector panels using these indexed reflections.

To perform this, we manually positioned the quadrants to best-fit powder patterns recorded on the back detector, as the diffraction was too sparse to obtain a good fit by rotational autocorrelation. We then performed spot-finding on the back detector images for all X-ray events in runs 11–22 that had been successfully indexed on the front detector, and then indexed these back detector reflections using the crystal models derived from the front detector lattices. This yielded 12 313 images where it was possible to index at least one reflection on the back detector. We then performed per-image r.m.s.d. filtering, removing images with an r.m.s.d. over 1.5 times the inter-quartile range (Tukey’s rule of thumb; Tukey, 1977 ▸), leaving 9893 images. Per-image r.m.s.d. filtering mainly removed images with false spots found along streaks produced by jet diffraction, which were visible as a long spike on a varying radial axis.

Next, we refined the back detector geometry against this data set using the hierarchical protocol. We fixed the crystal models, which had been refined against front detector data, as we only had a few reflections per image on the back detector. Table 8 ▸ shows the r.m.s.d., which improves with each successive hierarchical level of metrology refinement. Focusing on the common set only, the r.m.s.d. decreased from 740.5 to 361.3 µm during metrology refinement, or slightly over three pixels. Owing to the large sample-to-detector distance, these reflections cover many more pixels on the back detector (the mean number of pixels per reflection on the front detector for these 9893 images is 3.5, but on the back detector it is 22.8), yielding an r.m.s.d. on the same fractional order of accuracy as for the front detector refinement (about half a pixel).

Fig. 5 ▸(*b*) shows the composite maximum of the images collected from runs 11–22 after metrology refinement, indicating the Miller indices of each powder ring. The average signal from these reflections is well correlated with the calculated intensities from PDB entry 4ow3 from Hattne *et al.* (2014 ▸) (Fig. 5 ▸ *c*). This indicates that the refined metrology would be sufficiently accurate to integrate data from these images. Notably, a 001 ring is present, even though 001 is systematically absent in space group *P*6_1_22. We speculate that this arises from surface effects in small microcrystals with a high surface area to volume ratio. In any case there is some type of disorder that breaks the perfect 6_1_ screw symmetry of the crystal.

## 6. Ensemble refinement and crystal isomorphism   

**Figure 6:**
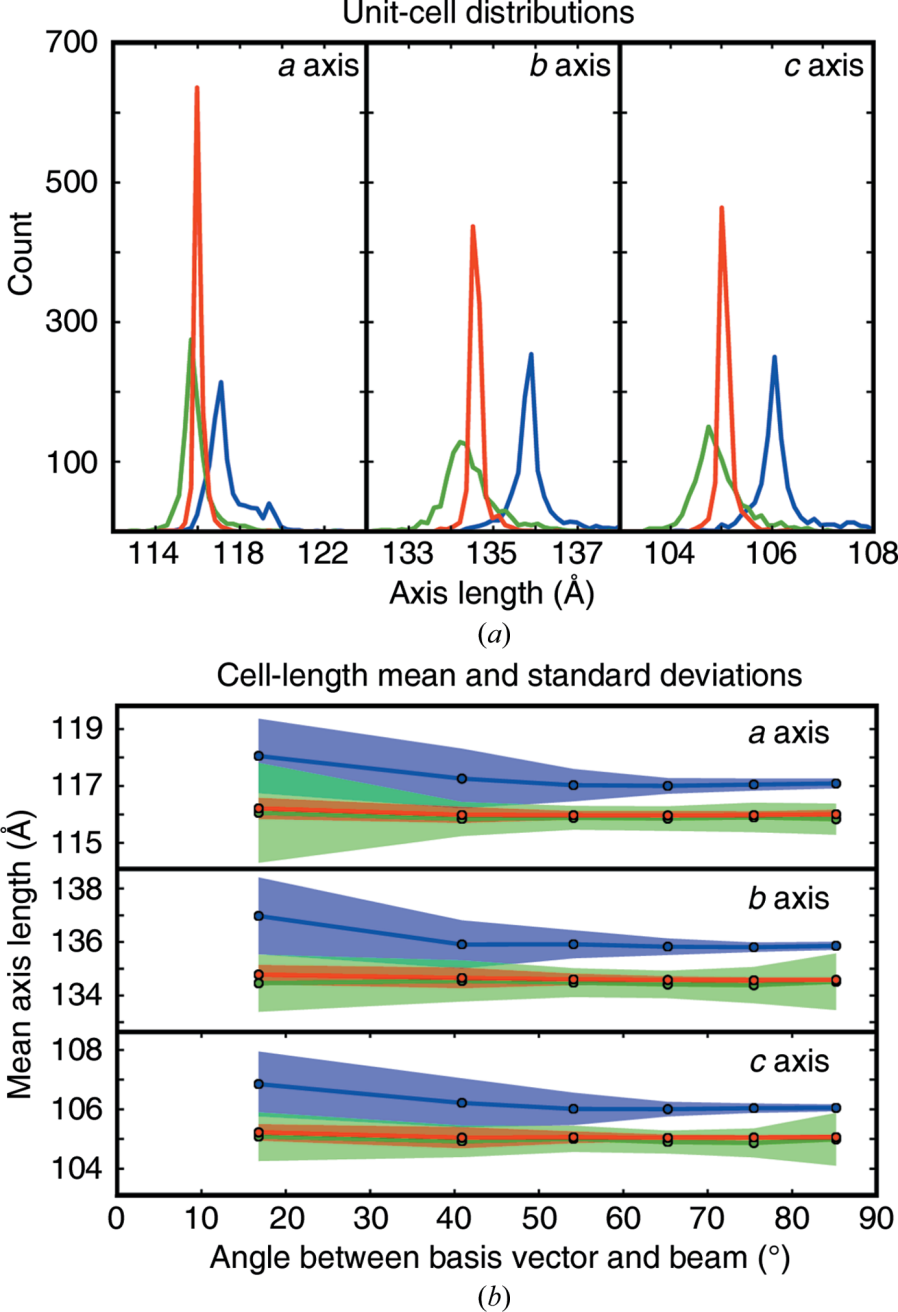
(*a*) Unit-cell histograms of 967 Cry3a lattices. The distribution of *a*, *b* and *c* axis lengths is shown. Blue: lattices were indexed and refined separately with no refinement of a detector model. Green: the same crystals after the refinement of 967 crystal models and 967 separate detector models. Red: the same except that the 967 crystal models were refined against a single detector model. (*b*) The cell-axis lengths from (*a*) were binned according to the angle of the axis with the beam vector. Bin widths are chosen such that the solid angle subtended within each bin is the same (a basis vector making an angle ρ with the beam will be in bin *i* satisfying the condition cos^−1^[1 − (*i*/6)] ≤ ρ < cos^−1^{1 − [(*i* + 1)/6]}, 0 ≤ *i* ≤ 5). The mean basis vector length for the *a*, *b* and *c* axes is plotted as a line, with one point for each bin at the bin center. The standard deviation among the values is shaded above and below the line. Colors are as in (*a*). The crystals rotated on average 0.25 and 0.23° during refinement using multiple or single detector models, respectively. The difference between the final models for multiple and single detector models was 0.04°.

Having optimized the detector models (§[Sec sec3]3), we now focus on the improvement of the crystal model, which consists of the crystal orientation and the unit-cell parameters. It has been widely observed in serial crystallography that within the ensemble of crystals merged together into a single data set, the unit-cell parameters exhibit an unusually broad distribution, far beyond that experienced in single-crystal work. Fig. 6 ▸(*a*), illustrates, for example, that the *a* axis of Cry3a in space group *C*222_1_ apparently varies from 116 to 120 Å (blue trace). This result was calculated by evaluating 1000 Cry3a patterns from run 4. Beginning with the previously refined front detector model determined from our thermolysin data, we indexed each Cry3a crystal lattice and refined the crystal parameters (unit cell and orientation) using traditional nonlinear least-squares refinement so as to best fit the reflections on each individual image. After refinement, 33 images with high r.m.s.d. were removed, giving a final data set consisting of 967 patterns.

It is critical to understand whether the apparent spread in unit-cell parameters represents true physical variation or simply an inability to measure the cell accurately. Indeed, if crystal cell lengths truly vary on the order of 3%, for example owing to differing hydration conditions (Russi *et al.*, 2011 ▸), it would challenge our ability to merge the diffraction patterns, since the structure-factor intensities from such a plastic ensemble would have too much variation to be usefully merged (Crick & Magdoff, 1956 ▸). We hypothesized instead that our widely distributed unit-cell measurements are a consequence of collecting data in still shots. In contrast to goniometer-based rotation experiments, where the reciprocal lattice is well sampled in all directions, still shots only sample reciprocal space to a limited depth along the direction of the incident beam. Therefore, if the *a* axis of an orthorhombic crystal is oriented along the beam, the *a* parameter should have a greater uncertainty than the *b* or *c* parameters. We chose an orthorhombic crystal form (Cry3a) to clearly test this supposition. Fig. 6 ▸(*b*) supports the hypothesis, showing that measurements of cell axes aligned with the beam have a much greater variation than cell axes oriented orthogonal to the beam (blue distributions).

We next asked whether it was possible to correct the biased distribution of unit cells with either of two protocols. In the first protocol, which did not provide a solution, we re-refined the unit-cell and orientation parameters, and also allowed the detector position (sample-to-detector distance and *xy* translation in the detector plane) to vary independently for each image. The resulting distributions are plotted in green (Fig. 6 ▸ and Supplementary Table S1). The unit-cell lengths were generally shorter, suggesting that the refined detector distance provides a better model (the sample-to-detector distance decreased from 111.00 mm to an average of 109.70 ± 0.64 mm). However, the artificially wide variation in unit-cell lengths did not improve; rather, it worsened slightly for cell axes oriented orthogonally to the beam. Unfortunately, we believe that this is the method that has been used historically for most serial crystallography work to date; at least, it is the approach taken by our program *cctbx.xfel* until this investigation.

In the second protocol, we sought to allow the detector to refine to its optimal position, but without allowing the detector model complete independence for every image. To this end, we performed joint refinement in which a single detector model was simultaneously refined against the ensemble of all crystal models in the data set. No explicit restraints were placed on the unit-cell parameters. The results (Fig. 6 ▸, red models) exhibit a similar decrease in unit-cell lengths (with concurrent shortening of the detector distance to 109.82 mm), but now the unit-cell standard deviations become much tighter than in the starting model. At least in this serial crystallo­graphy case, the apparent non-isomorphism of crystals seems to be an accuracy issue, which can be corrected by the joint refinement of the crystal ensemble against a single detector model, a new protocol that is enabled by the sparse-algebra LevMar technique outlined above.

## 7. Time-dependent ensemble refinement of the entire experiment   

From §[Sec sec6]6, it is clear that serial crystallography data sets can be acutely sensitive to experimental geometry, to the point that a nonphysical standard deviation of the crystal-to-detector distance of only 0.64 mm produces a noticeable and deleterious effect on the lattice parameters. Other literature results have also highlighted the need for precise distance determination, including a paper by Nass *et al.* (2016 ▸) that presents data where optimal unit-cell distributions and data-quality metrics can only be obtained by accounting for a series of distance shifts spanning 0.62 mm that occurred over a period of 3 days. Indeed, data-modeling software serves a decisive role in cases where it is not feasible to experimentally measure precise sample-to-detector distances. Our L785 thermolysin study provides one such example: the detector position is known from motor encoders, but the sample position can readily change depending on how the electrokinetic sample injector (Sierra *et al.*, 2012 ▸) is inserted and the flow characteristics of the liquid jet at any given point in time.

To investigate the possibility of a time dependence in the sample-to-detector distance parameter in our experiment, we split the data chronologically into runs. We then further sub­divided each run into a series of chronological batches each containing ∼3000–4500 images. For each batch, we performed joint refinement of a single detector model (freely refining the overall distance and the *xy* shift in the detector plane) against the ensemble of all crystal models in the batch. We then recomputed the mosaic estimates for each crystal (Sauter *et al.*, 2014 ▸), used these models to predict Bragg reflections, and integrated the Bragg intensities.

**Figure 7:**
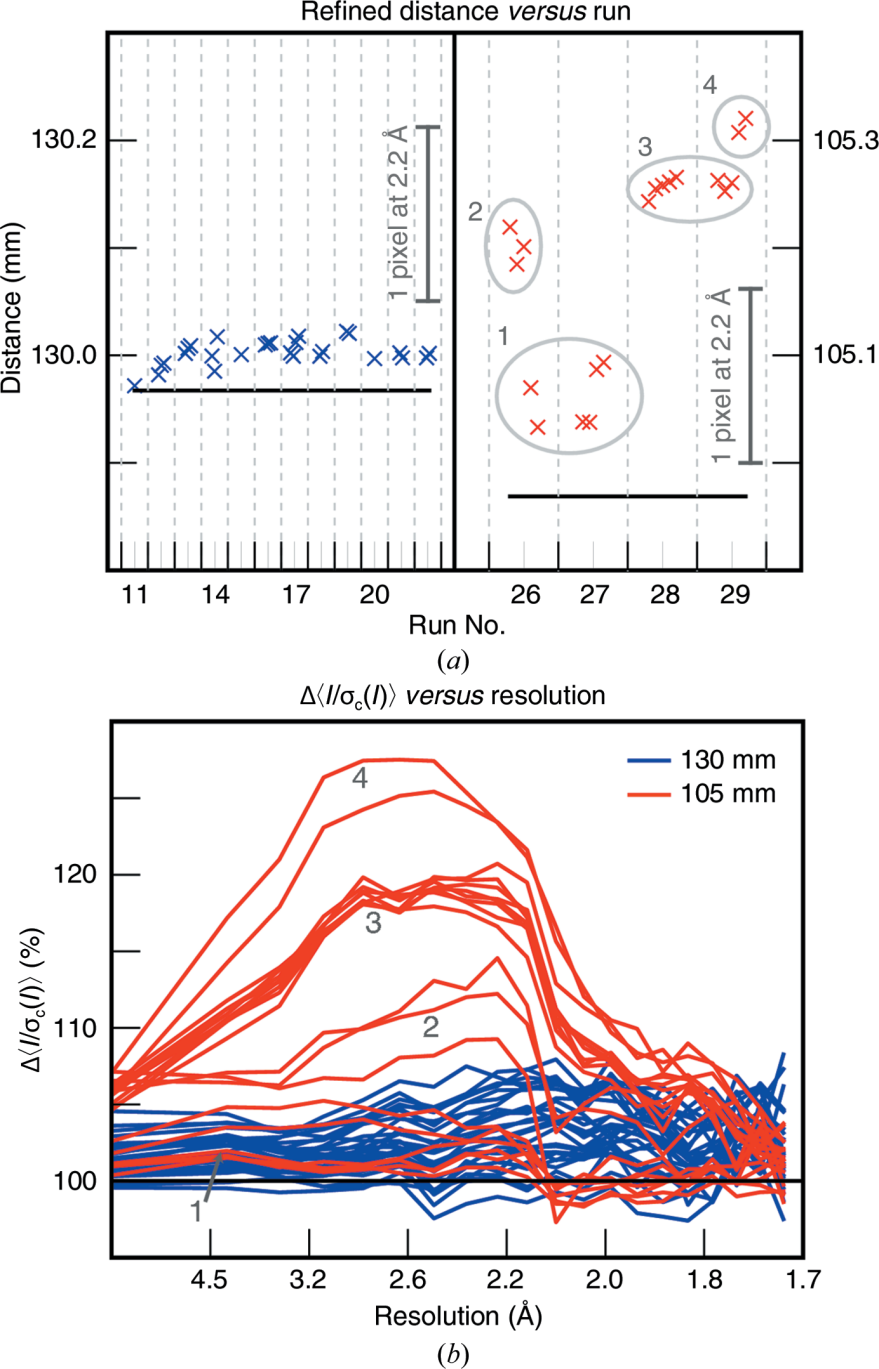
Time-dependent ensemble refinement and integration results. All lattices from two groups of data on the front detector (collected at distances of 130 and 105 mm) were separated chronologically into runs and then subdivided chronologically into batches of 3000–4500 images. The detector position and crystal models were refined for each batch separately and the data were then integrated. (*a*) Refined detector distances for each batch plotted *versus* run number. Black line: starting detector distance before refinement. The change in detector distance needed to effect a one-pixel radial shift for a reflection at 2.2 Å is shown as a vertical gray bar. (*b*) Percent change in signal strength 〈*I*/σ_c_(*I*)〉 [σ_c_(*I*) refers to the counting error, or the uncorrected error from integration summation] after per-run ensemble refinement. The mean resolution-binned signal after refinement for each batch was divided by the mean resolution-binned signal before refinement for that batch. Each line represents one batch. Percent change in signal is reported as a function of resolution. The numbered circles in (*a*) group together batches that have similar refined distances. These group numbers are also used to label the batches in (*b*).

Fig. 7 ▸(*a*) shows the resulting batchwise distance determinations. Baseline distance values are also indicated. For the first group (runs 11–22), the baseline is the direct result of metrology refinement from expanding-mode refinement (§[Sec sec3.6]3.6), 129.97 mm, based on the 3000 best-diffracting images. We see that time-dependent ensemble refinement produces slightly larger distance estimates, averaging 130.00 ± 0.01 mm. The cause of this increase is unknown, but we speculate that it could result from the time-dependent batches having fewer reflections in the detector corners compared with the analysis performed in §[Sec sec3.6]3.6 using a subsample of images with the most reflections in the corners. (As mentioned previously, a slight systematic radial misprediction of reflections may cause geometry refinement to be slightly resolution-dependent.) For runs 26–29, the baseline distance of 104.97 mm was produced by subtracting the motor-encoder offset (25 mm) for these runs from the calibrated distance in §[Sec sec3.6]3.6. It is clear that the distance values for runs 26–29 differ substantially (0.1–0.3 mm) from the baseline, and moreover that there is a 0.2 mm variability over the 36 min period, changing even within the duration of a single run.^3^ We assume that the underlying cause is that the flow direction of the liquid jet from the electrokinetic injector changed continually over time.

We expected that correctly accounting for our time-varying sample-to-detector distance would lead to improved Bragg spot predictions, as well as improvements in the data quality similar to those seen by Nass *et al.* (2016 ▸). As a data-quality metric (Fig. 7 ▸ *b*) we chose the signal strength, 〈*I*/σ_c_(*I*)〉, where *I* is the integrated intensity of a measurement, σ_c_(*I*) is the uncertainty in the measurement attributable to photon-counting statistics (Leslie, 2006 ▸) and 〈〉 represents the mean over all measurements of all Miller indices. For each batch, we compared the resolution-binned 〈*I*/σ_c_(*I*)〉 averages with the Bragg spots predicted from either the batchwise or the baseline distance values. Each line represents the resolution-dependent percent change in signal strength for a single batch. Runs 11–22 (distance 130 mm) exhibit a modest increase in signal of around 2–3%. However, runs 26–29 (distance 105 mm), especially runs 28–29, exhibit an appreciable increase in signal strength, up to 25% higher at mid-resolution for the last two batches, where the sample position moved 55 µm mid-run. Thus, time-dependent, batchwise ensemble refinement appears to offer the possibility of detecting and correcting changes in the experimental model on timescales not previously understood to present a challenge for data quality.

## 8. Merging and error models   

Table 9 ▸ shows a comparison between the originally published thermolysin structure (PDB entry 4tnl) and the data reprocessed in this work, modeled using the expanding-mode metrology in §[Sec sec3.6]3.6. Data were scaled and merged using four alternate protocols differing in the use of post-refinement (Sauter, 2015 ▸), the use of time-dependent ensemble refinement of the detector distance (§[Sec sec7]7) and the choice of error model used to adjust the estimated errors of the integrated, merged intensities. Supplementary Tables S2–S5 give detailed statistics for the four resulting data sets.

Critically, interpreting signal quality is highly dependent on the error model, *i.e.* how the uncertainties in the measured reflection intensities are treated. During integration, we determine a baseline estimate of the error from photon-counting statistics (Leslie, 2006 ▸), referred to above as σ_c_(*I*), with this being the only source of uncertainty that is readily quantified. Other sources of error from detector calibration, partiality correction, crystal orientation and cell dimensions, among many others, are much more difficult to estimate and therefore propagate. However, because the errors from counting statistics derived from integration represent only a small part of the overall experimental uncertainty, it is imperative to inflate the error determined from counting statistics to better represent the error seen in the sample.

One such treatment, Ha14, was proposed in Hattne *et al.* (2014 ▸). In that work, we considered the distribution of all intensity measurements produced on a single serial crystallo­graphy still shot. Familiar principles would lead us to believe that the measurements would form an exponentially decreasing distribution, as originally discussed by Wilson (1949 ▸). However, since most (if not all) spots on a still shot are partials, and since the degree of partiality is not known *a priori*, the prediction of still-shot spots puts us in a difficult position. In order to predict all of the diffracted Bragg spots, we need to slightly overmodel the mosaic parameters (Sauter *et al.*, 2014 ▸). As a consequence, many of the predicted reflections will not contain any photons, and the process of integration simply produces noise, which has a Gaussian distribution rather than an exponential one. A recent paper (Sharma *et al.*, 2017 ▸) shows this explicitly. In the Ha14 approach, we look at the distribution of the quantity *I*/σ_c_(*I*) over all measurements on the image. The negative values of *I*/σ_c_(*I*) are assumed to form the lower half of a Gaussian distribution (with a mean of zero) for which we determine the standard deviation σ_neg_. With the assumption that the negative measurements represent the background noise level, we therefore use σ_neg_ as a constant, dimensionless multiplicative factor to inflate the photon-counting uncertainty, giving a new uncertainty estimate for each measurement,
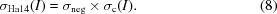



Separately, we have adapted the error model employed by *SCALA* (Evans, 2006 ▸, 2011 ▸), Ev11. This model expresses the uncertainty in terms of three refined parameters: SdFac (a multiplicative factor), SdB (a factor proportional to reflection intensity) and SdAdd (a factor proportional to intensity squared), as formulated in the following:
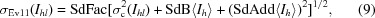
where *I_hl_* is a single reflection measurement of Miller index *h*, σ_c_^2^(*I_hl_*) is the error in that measurement from integration summation, 〈*I_h_*〉 is the mean of all measurements of *h* and σ_Ev11_(*I_hl_*) is the corrected error in a single reflection measurement as treated by Evans (2011 ▸).

Comparing Ha14 and Ev11, these two models produce very different error estimates of the error when propagated to the merged intensities. It is likely that Ha14 substantially underestimates the error in the data, producing overall *I*/σ_Ha14_(*I*) estimates of ∼300. The overall *I*/σ_Ev11_(*I*) estimates of ∼30 are more reasonable based on what is known generally about error in crystallographic experiments (Diederichs, 2010 ▸). Ha14 was never intended as a final description of the error model; however, Ev11, while giving reasonable numerical error ranges, does not account for the propagation of errors from partiality corrections that are inherently needed for serial crystallography stills.

## 9. Discussion   

*DIALS* provides a general representation of complex experimental geometry that we have shown here to be suitable for serial crystallography. We demonstrated this by refining the metrology of the CSPAD detector at LCLS using a thermolysin data set. Firstly, we used a rotational autocorrelation approach to position the quadrant locations using a virtual powder pattern. We then treated all 32 CSPAD sensors as independent panels using an expanding approach to metrology refinement, first refining the inner tiles and then progressively adding tiles in increasing resolution shells. After refining the metrology, we show a large improvement of the r.m.s.d. of the observed *versus* predicted spot locations (from 157.9 to 60.1 µm, or about half a pixel given the CSPAD pixel size of 110 µm). We observed a further improvement after reindexing the images using the new metrology and re-refining in cycles until convergence (giving a final r.m.s.d. of 50.1 µm after two cycles).

In order for joint parameter refinement to be practical using thousands of crystal models with a multi-panel detector, we implemented a nonlinear least-squares approach using sparse-matrix algorithms to efficiently solve the normal equations required for computing step sizes. This allows us to use a single joint target function instead of alternating between crystal and detector models, as had been performed previously.

We were also able to use the crystal orientation matrices from lattices observed on one CSPAD to refine the metrology of a second CSPAD detector positioned 2.5 m from the sample-interaction region, demonstrating the general approach to geometry refinement used by *DIALS*. We can combine detectors, refine overall detector distance and panel positions simultaneously, and handle detectors with any number of segments in arbitrary orientations.

When joint refinement is applied to the ensemble of all crystals used for a data set, it has the added benefit of improving the accuracy of unit-cell length estimation for axes closely aligned with the beam, which are difficult to measure from still images. Serial crystallography data sets typically exhibit a broad distribution of unit-cell parameters derived from indexing, presumably reflecting several factors, including an inability to accurately model the detector position, crystal parameters and beam parameters, as well as true non-isomorphism among crystals. Ensemble refinement offers a method for removing several types of modeling uncertainty, producing a clearer picture of the inherent non-isomorphism of the crystals, which in the case of our thermolysin data was very low. We note that worries about non-isomorphism in serial crystallography have been broadly expressed, and our results appear to alleviate some of the concern.

Ensuring a correct detector position is vital for maximizing the number of integrated patterns and correcting for asymmetry in the unit-cell distributions (Hattne *et al.*, 2014 ▸; Nass *et al.*, 2016 ▸). It is expected that the sample position can and will vary slightly over time during data collection, and even drift from its original position, as scientists replace the sample, recalibrate equipment positions or simply bump instruments. To this end, we tried to correct for systematic changes in detector position over time by applying a time-dependent ensemble refinement approach to improving the integrated data quality across an entire experiment. We batched the data into granular time intervals and re-refined the detector position, crystal models and mosaic estimates within each batch, using the new models to predict and integrate the Bragg spots. This gave a slight improvement in signal strength for data collected at the same time that the detector metrology calibration data were collected (130 mm detector distance). When the detector was moved to 105 mm, re-refining the detector position for each batch further improved the integrated signal strength.

After metrology refinement, the improved tile positions can be reused during later data collection by either specifying the refined geometry file directly in *DIALS* or converting it to LCLS geometry format for general users of non-*DIALS* software (Brewster *et al.*, 2016 ▸). However, changes in detector position can require tile-position re-refinement. Small, resolution-dependent systematic errors in spot prediction (§[Sec sec3.6]3.6) can be absorbed by local tile movements, so potentially users should always re-refine the detector metrology when moving the detector.

We merged the data using four different protocols. The best-practice method, as measured by the anomalous peak height for the Zn atom of 74.0σ, included post-refinement, time-dependent ensemble refinement and the Ev11 error model. Removing post-refinement, removing time-dependent ensemble refinement or using the Ha14 error model each resulted in lower anomalous peak heights (42.6, 69.4 and 44.6σ, respectively). We recommend that software users experiment with these algorithm choices when analyzing future XFEL data sets. Scripts to do so have been made available (see §[Sec sec10]10).

## 10. Data and software availability   

Raw thermolysin data files for experiment L785 are available from the Coherent X-ray Imaging Data Bank as deposition 81 (http://www.cxidb.org/id-81.html). Jupyter notebooks that reproduce all seven figures in this work are available at https://github.com/phyy-nx/dials_refinement_brewster2018. All of the metrology refinement, integration and merging approaches outlined here are implemented in the software package *DIALS*, which is publicly available at https://dials.github.io. Documentation regarding metrology refinement and XFEL processing in *DIALS* is also available in Brewster *et al.* (2016 ▸) and on the *cctbx.xfel* wiki page at http://cci.lbl.gov/xfel.

## Figures and Tables

**Table 1:** Data sets Sample names and LCLS proposal numbers are given for the two data sets used in this work, as well as the total number of shots collected, the detector distances at which the data were collected and the incident photon energy.

Sample	LCLS proposal No.	Published PDB code	Run Nos.	Collection time (min)	No. of shots	Front detector distance (mm)	Back detector distance (m)	Photon energy (keV)
Thermolysin	L785	4tnl	11–22	71	503605	130	2.5	9.75
			26–29	36	256505	105	2.5	9.75
Cry3A toxin	LS02	4qx0	2–4	7	49453	111	2.5	8.50
			5	5	38808	181	2.5	8.50
			6–7, 10–12	41	292479	166	2.5	8.50

**Table 2:** Results of alignment by rotational autocorrelation Runs: a weak run (22), a strong run (14) and a many-run composite image (runs 13–22). For the single-angle method and the multi-angle method, for each quadrant the pixel offset needed to move the quadrant to maximize the rotational autocorrelation CC and the CC at that position are listed.

		Single-angle method		Multi-angle method	
Run(s)	Quadrant	CC (%)	Quad offset (pixels)	CC (%)	Quad offset (pixels)
22	0	6.1	(3, 2)	16.4	(20, −20)
	1	8.0	(5, 2)	12.5	(−8, −13)
	2	6.3	(2, 1)	11.9	(−19, 6)
	3	6.5	(1, −5)	11.1	(11, 15)
14	0	21.5	(3, 2)	24.6	(3, 2)
	1	23.0	(5, 3)	27.0	(5, 3)
	2	25.8	(4, 0)	27.7	(4, 0)
	3	23.6	(0, −6)	26.5	(1, −5)
13–22	0	28.4	(3, 2)	32.5	(3, 2)
	1	32.6	(5, 3)	37.7	(5, 4)
	2	36.6	(4, 0)	41.5	(3, 0)
	3	33.9	(0, −6)	37.8	(0, −7)

**Table 3:** Refinement procedure (hierarchical mode) Level: the hierarchy level being refined. At the detector level, the basis frame of the entire detector is refined as a block. At the quadrant level, the four quadrant frames are independently refined. At the sensor level, the 32 individual sensors are independently refined. Sensors: which of the eight sensors per quadrant are refined (see Fig. 1 ▸). Fix: parameters that are not allowed to refine. Refine: parameters that are refined (note that all of the thousands of crystal cells and orientations are always simultaneously refined with the detector model). Constraints: how the parameters are constrained to change in concert. After the first refinement step, the distance parameter of the child frames being refined are all constrained to change by the same amount. This preserves a coplanar detector while refining the detector distance at each step.

Level	Sensors	Fix	Refine	Constraints
Detector	All	τ_1_, τ_2_, τ_3_	Distance, Shift1, Shift2	None
Quadrants	All	τ_1group1_, τ_2_, τ_3_	Distance, τ_1_, Shift1, Shift2	Coplanar
Sensors	All	τ_1group1_, τ_2_, τ_3_	Distance, τ_1_, Shift1, Shift2	Coplanar

**Table 4:** Refinement procedure (expanding mode) As Table 3 ▸, except that not all sensors are refined at each level. Instead, sensors are added in order of increasing radial distance from the detector center. For example, in the first refinement step reflections from sensor 1 of each quadrant are used to refine the detector as a whole. In the second refinement step, the same reflections are used to refine the quadrants individually. In the third, sensors 0 and 1 from the four quadrants are refined individually, and so forth until all 32 sensors are refined in the last step.

Level	Sensors	Fix	Refine	Constraints
Detector	1	τ_1_, τ_2_, τ_3_	Distance, Shift1, Shift2	None
Quadrants	1	τ_1group1_, τ_2_, τ_3_	Distance, τ_1_, Shift1, Shift2	Coplanar
Sensors	1, 0	τ_1group1_, τ_2_, τ_3_	Distance, τ_1_, Shift1, Shift2	Coplanar
Sensors	1, 0, 7	τ_1group1_, τ_2_, τ_3_	Distance, τ_1_, Shift1, Shift2	Coplanar
Sensors	1, 0, 7, 3	τ_1group1_, τ_2_, τ_3_	Distance, τ_1_, Shift1, Shift2	Coplanar
Sensors	1, 0, 7, 3, 2	τ_1group1_, τ_2_, τ_3_	Distance, τ_1_, Shift1, Shift2	Coplanar
Sensors	1, 0, 7, 3, 2, 6	τ_1group1_, τ_2_, τ_3_	Distance, τ_1_, Shift1, Shift2	Coplanar
Sensors	1, 0, 7, 3, 2, 6, 4	τ_1group1_, τ_2_, τ_3_	Distance, τ_1_, Shift1, Shift2	Coplanar
Sensors	1, 0, 7, 3, 2, 6, 4, 5	τ_1group1_, τ_2_, τ_3_	Distance, τ_1_, Shift1, Shift2	Coplanar

**Table 5:** R.m.s.d. of reflections on the front detector R.m.s.d.s of the observations *versus* the predictions are listed at each cycle of indexing and refinement for each mode, hierarchical and expanding. Step: stage in refinement procedure. Initial: r.m.s.d. of indexed reflections before refinement. Cycle 1: r.m.s.d. of reflections after refinement. Cycles 2–4: r.m.s.d.s after reindexing using the metrology of the previous cycle and re-refining the same set of images. The overall r.m.s.d. is listed, which is the r.m.s.d. of all reflections in the data set for the cycle. The ‘common set’ r.m.s.d. is also listed, which is the r.m.s.d. of reflections that were indexed in every data set between both modes (218 954 reflections).

	Hierarchical				Expanding			
			R.m.s.d. (µm)				R.m.s.d. (µm)	
Step	No. of images	No. of reflections	Overall	Common set	No. of images	No. of reflections	Overall	Common set
Initial	3000	700222	221.0	157.9	3000	700222	221.0	157.9
Cycle 1	2999	621516	138.6	62.9	3000	580093	129.4	60.1
Cycle 2	2727	383446	81.5	56.5	2701	328309	62.3	50.1
Cycle 3	2705	361339	76.0	55.4	2684	302271	54.4	47.9
Cycle 4	2712	355910	73.6	54.8	2709	298874	51.6	48.0

**Table 6:** Magnitudes of metrology changes Changes after each cycle of indexing and refinement using the expanding method are shown. Cycle 1: result after refining initial indexing solutions. Cycles 2–4: results after reindexing using the metrology of the previous cycle and re-refining using the expanding method. Shift: mean ± standard deviation of the magnitude of shifts in the *xy* plane (orthogonal to the beam vector). As an example, during cycle 1 the four quadrants moved 138 ± 111 µm on average. *z* Offsets: shifts along the *z* axis (equivalent to the detector distance). As the detector is constrained to be coplanar, all groups move the same amount. τ_1_: mean ± standard deviation of the rotation around the *z* axis. All values are relative to the parent frame.

	Level	Shift (µm)	*z* Offsets (µm)	τ_1_ (°)
Cycle 1	Detector	56.3 ± 0.0	99.3	0.0 ± 0.0
	Quadrants	138.3 ± 110.7	−118.1	0.2 ± 0.2
	Sensors	53.7 ± 53.5	37.2	0.2 ± 0.1
Cycle 2	Detector	0.4 ± 0.0	−42.8	0.0 ± 0.0
	Quadrants	3.8 ± 1.5	−18.0	0.0 ± 0.0
	Sensors	9.8 ± 6.3	71.0	0.0 ± 0.0
Cycle 3	Detector	0.1 ± 0.0	−84.0	0.0 ± 0.0
	Quadrants	1.6 ± 0.5	3.6	0.0 ± 0.0
	Sensors	11.1 ± 6.1	86.0	0.0 ± 0.0
Cycle 4	Detector	0.2 ± 0.0	−92.2	0.0 ± 0.0
	Quadrants	1.5 ± 0.7	3.3	0.0 ± 0.0
	Sensors	9.0 ± 6.3	90.9	0.0 ± 0.0

**Table 7:** LevMar memory and run time Order of growth of memory requirements and run time of LevMar and sparse LevMar techniques as a function of the number of images. The exponent and coefficient of determination (*R* ^2^) of a power-function fit to the data is shown for the LevMar and sparse LevMar run times from Fig. 4 ▸(*a*) and for each of the plots in Fig. 4 ▸(*d*). The *R* ^2^ value gives a measure of the fit of the power function given the exponent.

	Exponent	*R* ^2^ (%)
Normal matrix square size	1.89	99.9
Normal matrix nonzeros	1.01	100.0
Cholesky factor nonzeros	0.95	100.0
LevMar time	2.45	98.5
Sparse LevMar time	1.13	98.6

**Table 8:** R.m.s.d. of reflections on the back detector Each row shows the overall and common-set r.m.s.d.s after refining at the listed hierarchy level.

Step	No. of reflections	R.m.s.d., overall (µm)	R.m.s.d., common set (µm)
Initial	11381	926.4	740.5
Level 0 (detector)	10820	797.9	683.0
Level 1 (quadrants)	10546	630.1	496.4
Level 2 (sensors)	9896	361.3	361.3

**Table 9:** Summary of statistics for post-refinement and merging of thermolysin data The original processing results from Kern *et al.* (2014 ▸) are compared with the processing in this work. Four merging protocols are presented, differing in whether post-refinement was applied, whether time-dependent ensemble refinement was applied and in the error model used. Values in parentheses are for the highest resolution bin.

Data set	Kern *et al.* (2014 ▸)	1	2	3	4
Post-refinement	No	Yes	No	Yes	Yes
Time-dependent ensemble refinement	No	Yes	Yes	No	Yes
Error model	Ha14	Ev11	Ev11	Ev11	Ha14
Measurement time (min)	107	107	107	107	107
Shots	757546	757546	757546	757546	757546
Lattices indexed and integrated	125800	166250	166250	166250	166250
Lattices merged	120408	164585	164612	165954	164556
Resolution range (Å)	34.27–1.80 (1.86–1.80)	34.27–1.80 (1.86–1.80)	34.27–1.80 (1.86–1.80)	34.27–1.80 (1.86–1.80)	34.27–1.80 (1.86–1.80)
Space group	*P*6_1_22	*P*6_1_22	*P*6_1_22	*P*6_1_22	*P*6_1_22
*a*, *b*, *c* (Å)	93.0, 93.0, 130.4	93.2, 93.2, 130.8	93.2, 93.2, 130.8	93.2, 93.2, 130.6	93.2, 93.2, 130.8
Multiplicity	1468 (15)	1178 (500)	1548 (783)	1132 (449)	1178 (500)
Completeness (%)	99.7 (98.6)	100.0 (100.0)	100.0 (100.0)	100.0 (100.0)	100.0 (100.0)
〈*I*/σ(*I*)〉†	71.7 (4.1)	32.4 (10.3)	32.3 (8.0)	28.1 (8.6)	311.3 (30.2)
CC_1/2_ (%)	97.8 (21.2)	100.0 (85.6)	99.6 (81.9)	99.9 (81.1)	99.8 (69.1)
Anomalous difference map peak heights‡ (σ)					
Zn^2+^	18.1	74.0	42.6	69.3	44.6
Ca^2+^ 1	4.7	17.1	10.7	15.9	9.7
Ca^2+^ 2	4.0	12.0	7.2	12.3	7.4
Ca^2+^ 3	3.3	15.3	9.9	13.3	9.8
Ca^2+^ 4	2.4	16.1	10.6	14.8	10.5
Average Ca^2+^	3.6	15.1	9.6	14.1	9.4

† This is the mean intensity over either σ_Ha14_(*I*) or σ_Ev11_(*I*) (see text for details).

‡ A second zinc site, as shown in Uervirojnangkoorn *et al.* (2015 ▸), is observable in our data as well but was not modeled in this work.

## Footnotes

1 More complex panels, such as curved or nonrectangular chips, can be represented in *DIALS* given an appropriate pixel-to-millimetre implementation. At present, the pixels need to be addressable using two dimensions, but the software can be extended to triangular or hexagonal pixel shapes should the need arise. In the case of the CSPAD all panels have the same pixel size, but this need not be true for other detectors represented by models in *DIALS*.

2 We developed a prototype using explicit second derivatives but found this to be a time-consuming process that does not lend itself well to rapid software development and algorithm improvement.

3 For runs 11–22 the mean r.m.s.d. for all of the batches after ensemble refinement was 40.5 µm. For runs 26–29 it was 73.6 µm. Why runs 26–29 have a larger r.m.s.d. is unknown. We attempted ensemble refinement of each batch again, but instead of only refining detector distance and *xy* shift, we also refined all of the sensor positions (see Table 4 ▸, last row). This ensemble refinement did not further improve the r.m.s.d.s for runs 26–29 (data not shown).
